# Genome Sequence Analysis of Dengue Virus 1 Isolated in Key West, Florida

**DOI:** 10.1371/journal.pone.0074582

**Published:** 2013-09-30

**Authors:** Dongyoung Shin, Stephanie L. Richards, Barry W. Alto, David J. Bettinardi, Chelsea T. Smartt

**Affiliations:** 1 Department of Entomology and Nematology, University of Florida, Vero Beach, Florida, United States of America; 2 Department of Health Education and Promotion, College of Health and Human Performance, East Carolina University, Greenville, North Carolina, United States of America; 3 Department of Molecular and Cellular Biology, University of Illinois, Urbana, Illinois, United States of America; Thomas Jefferson University, United States of America

## Abstract

Dengue virus (DENV) is transmitted to humans through the bite of mosquitoes. In November 2010, a dengue outbreak was reported in Monroe County in southern Florida (FL), including greater than 20 confirmed human cases. The virus collected from the human cases was verified as DENV serotype 1 (DENV-1) and one isolate was provided for sequence analysis. RNA was extracted from the DENV-1 isolate and was used in reverse transcription polymerase chain reaction (RT-PCR) to amplify PCR fragments to sequence. Nucleic acid primers were designed to generate overlapping PCR fragments that covered the entire genome. The DENV-1 isolate found in Key West (KW), FL was sequenced for whole genome characterization. Sequence assembly, Genbank searches, and recombination analyses were performed to verify the identity of the genome sequences and to determine percent similarity to known DENV-1 sequences. We show that the KW DENV-1 strain is 99% identical to Nicaraguan and Mexican DENV-1 strains. Phylogenetic and recombination analyses suggest that the DENV-1 isolated in KW originated from Nicaragua (NI) and the KW strain may circulate in KW. Also, recombination analysis results detected recombination events in the KW strain compared to DENV-1 strains from Puerto Rico. We evaluate the relative growth of KW strain of DENV-1 compared to other dengue viruses to determine whether the underlying genetics of the strain is associated with a replicative advantage, an important consideration since local transmission of DENV may result because domestic tourism can spread DENVs.

## Introduction

Dengue virus (DENV, Family *Flaviviridae*, genus *Flavivirus*) is transmitted to humans through mosquito bites and dengue is epidemic and endemic in tropical and subtropical regions [1]. Dengue virus is a globally important pathogen, annually infecting approximately 50 million people in 100 countries, including The Americas, Asia, Middle East, and Africa [2]. Dengue virus exists in four genetically distinct serotypes (1–4) and serious health conditions can occur when there is sequential or co-infection of more than one serotype, i.e. dengue hemorrhagic fever and dengue shock syndrome [3] [4].

The Americas have seen an increase in human cases of dengue in recent years [2] [5] where all four DENV serotypes are prevalent [2] [6]. Generally, DENV-1 and DENV- 2 were the most frequently reported serotypes during the 1990s and, recently, DENV- 2 and DENV- 3 were the most frequent serotypes reported from 2000–2007 [2]. Nicaragua (NI) and Mexico are dengue endemic areas and both are in the same Central America/Mexico sub-region, as defined by the Pan America Health Organization (PAHO) [7]. Dengue cases in the Central America/Mexico region showed an increase from 1980 to 2007 [2]. In NI, there were DENV-1 outbreaks from 2002–2005 and DENV-1 is currently still circulating in that region [8]. Puerto Rico (PR) is included in the Hispanic Caribbean region as defined by PAHO [7] and dengue outbreaks were reported from 1963–1998 [9]. After nearly a decade of no significant dengue outbreaks, an island wide outbreak occurred in PR in 2007. Although all four serotypes co-circulate in PR, DENV- 2 and DENV- 3 were the serotypes primarily detected during the outbreak in 2007 [10] [11].

The Central America/Mexico and Hispanic Caribbean regions are geographically close to the southern U.S. and there is a great deal of tourism between the U.S. and both regions. Traveler-imported cases can lead to large-scale epidemic transmission [12] [13] [14]. In 2005, there were 96 traveler-imported dengue cases in the U.S., including 12 cases in Florida [15]. Many DENV infections (DENV-1 and -3) were also observed on the international border of Texas and Mexico in 2005 [16]. Florida has a history of epidemic DENV transmission and recent cases were likely imported by tourism [17]. In 2009, from July-October, a focal dengue outbreak occurred in KW (Monroe County), Florida where 9/21 (43%) physician-submitted cases and 13/240 (5%) area residents surveyed were positive for DENV or DENV antibodies [18]. Two *Aedes aegypti* mosquito pools collected in KW from September-December 2009 were DENV-1-positive [17]. In 2010, another dengue outbreak occurred in KW with 24 locally acquired cases reported as of July 31, 2010 [17] [18]. Sporadic cases of dengue have continued to occur, focused in south Florida [19].

Both the yellow fever mosquito, *Aedes aegypti*, primary vector of DENV, and the Asian tiger mosquito, *Aedes albopictus*, are found in dengue endemic and epidemic regions. These *Aedes* spp. coexist in similar habitats in Florida [20] [21]. Though *Ae. aegypti* is the primary vector of DENV, *Ae. albopictus* has been a primary vector in some dengue epidemics in the U.S. [22]. South Florida has large urban populations of these *Aedes* spp. and human populations that lack immunity to DENV, hence the potential for recurrent dengue in this region is a significant threat. Dengue outbreaks are related to the rainy season, because mosquito density increases with rainfall and, hence, oviposition sites are highest [23] [24]. The same is true in Florida, where most dengue cases have occurred during the rainy season (May to October).

Recombination generates genetic variation and contributes to the evolution of viruses [25] [26]. South American and Cambodian DENV-1 strains showed recombination as strong evidence of the role of recombination in DENV-1 evolution [27] [28]. Virus recombination can occur without known reason or when a host (e.g. mosquito or human) is co-infected by different virus strains [26] [29]. Recombination of DENV identified by a sliding window analysis and applied to phylogenetic methods between different strains is a powerful approach used to improve our understanding of the evolutionary history of viruses. This approach can be used to: 1) identify the origin of the virus isolate, 2) define isolates showing genetic and geographic variation, and 3) determine the genetics of DENV associated with outbreaks in KW, Florida.

Despite more than 1,000 travel-associated dengue cases in the U.S. between 1996 and 2005 [30], cases of locally acquired dengue in the continental U.S. have been rare [31]. Puerto Rico is an unincorporated territory of the U.S. where dengue is endemic and travel between Puerto Rico and the U.S. mainland is common which may pose a risk for traveler-imported dengue cases. Yet, to our knowledge, dengue viruses from this region have not been associated with the establishment of endemic transmission in the continental U.S. Population dynamic theory suggests that populations that grow rapidly beyond small population sizes vulnerable to stochastic extinction have a greater probability of successful colonization of an empty site compared to populations with slower growth [32] [33]. This idea is applicable to virus infection in hosts and vector, e.g., some dengue viruses are known to have replicative advantage (more virulent) over others, contributing to displacement and disease emergence [34] [35].

This study used genome sequencing to characterize the Florida DENV-1 isolate implicated in the 2010 outbreak and compares the sequence with other known DENV sequences. We use a tissue-culture model system to evaluate the relative growth of DENV-1 isolate associated with endemic transmission in Florida [36] to DENV from Puerto Rico, part of a region associated with the highest number of travel-associated dengue cases in the U.S. [30]. We sought quantitative evidence that the Florida DENV-1 isolate has a relative growth advantage compared to other DENV that have likely been introduced due to traveler-imported dengue cases, but were not associated with endemic transmission. The current status of DENV in KW, Florida is discussed. This baseline knowledge improves our understanding of the underlying genetics and growth of an isolate of DENV associated with endemic transmission in the continental U.S.

## Results

### Comparison of the growth of DENV isolates

**Figure 1:**
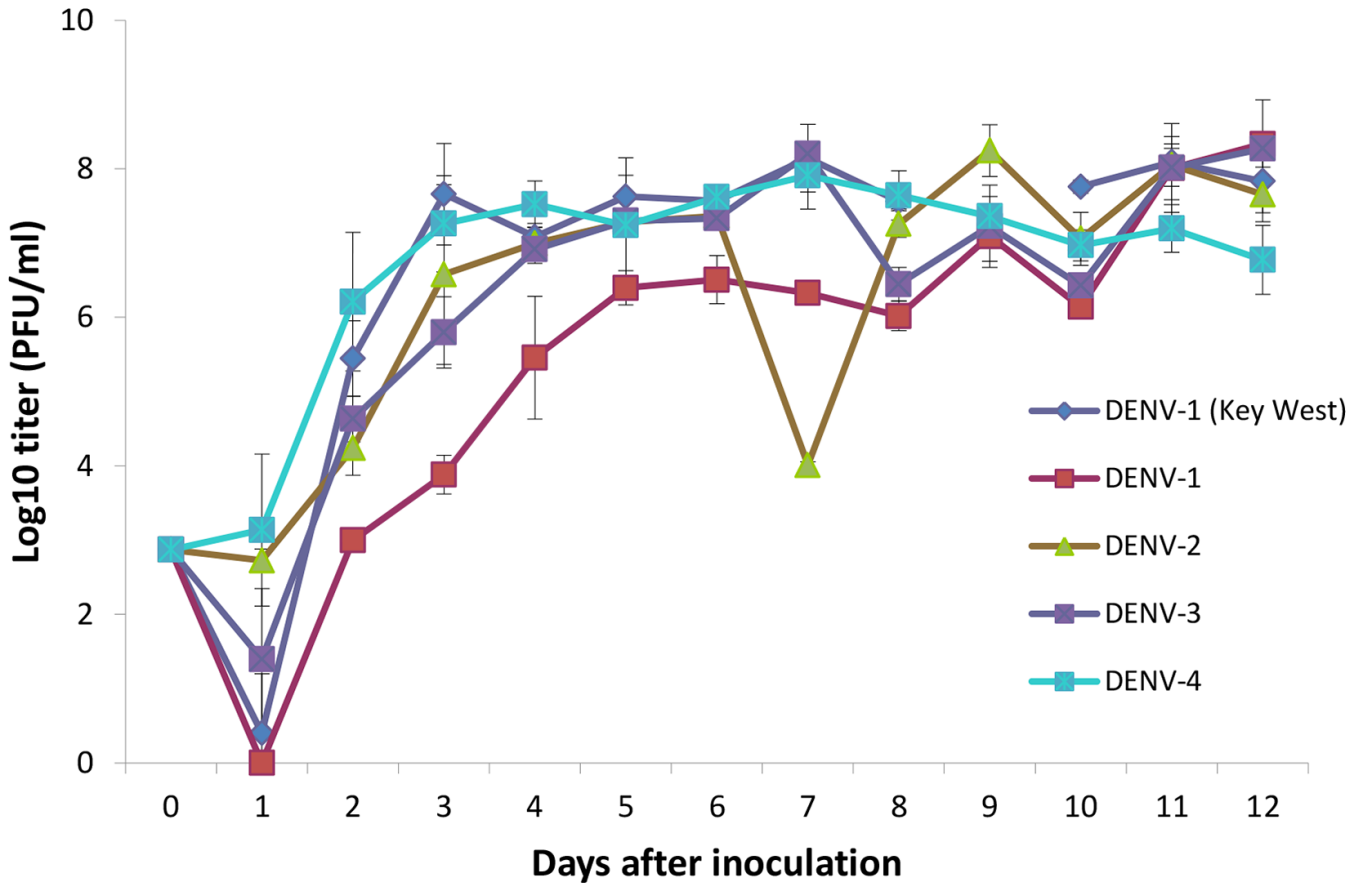
Dengue virus growth curves in Vero cells over a 12-1, PR DENV-2, PR DENV-3, PR DENV-4, and KW DENV-1. Standard deviation is calculated from replicate (N = 4) samples tested at each time point and each isolate showed significantly different growth rates (ANOVA, *p*<0.001).

Virus kinetics were compared between PR (DENV-1, -2, -3, -4) and KW (DENV-1) isolates in Vero cell since DENV is endemic in PR and there is potential for importation of human cases into FL (Figure 1). The analysis of variance (ANOVA) indicated that titers of DENV varied significantly between isolates and days post infection (dpi) during both the initial (i.e. 1–5 dpi) and later (i.e. >5 dpi) growth phases (Figure 1). We also observed a significant interaction between isolate and dpi for viral titers during both the initial and later growth phases (Figure 1). During the initial growth phase, titers were significantly highest (*P*<0.001) for DENV-1 from KW on 3 and 5 dpi, with the lowest titers observed in DENV-1 (PR) at 1 dpi (Figure 1). Analyses on the later growth phase showed titers were highest (*P*<0.001) in DENV-1 (PR) at 12 dpi (Figure 1). Overall, DENV-1 from KW had a significant replicative advantage over DENV-1 (PR) at eight of the 12 time points (*P*<0.001). DENV-1 from KW had equal or significantly greater growth than DENV-2 and DENV-3, but not DENV-4, at all but one of the time points in the initial growth phase (*P*<0.001). DENV-1 from KW had equal or significantly greater growth than DENV-2, DENV-3 and DENV-4 at all time points during the later growth phase (*P*<0.001).

### Genome sequence of KW DENV-1

The genome of the KW DENV-1 isolate was sequenced and the full length was 10,600 bp. The virus was amplified as 13 overlapping fragments by RT-PCR and sequenced directly. All of the sequenced fragments were assembled into a single contig of 10,600 bp with DNASTAR (Version 10.0.0). The entire assembled sequence was deposited in GenBank (Accession number JQ675358). The KW DENV-1 nucleotide sequence is best matched with NI DENV-1 strain, isolated in 2006 (FJ562104; NI2006). Pairwise nucleotide sequence alignment of the KW 2010 and NI 2006 isolates showed an identity of 99%, and 66 nucleotide changes throughout the genome (Table 1). Based on the sequence identity and nBlast total score, the NI and the Mexico (MX) strains from 2004 to 2008 were in the top 100 best-match sequences to the KW DENV-1 (data not shown). The KW DENV-1 amino acid sequence is best matched with a NI strain from 2005 (FJ850114; NI2005) with 99% identity.

**Table 1:** Percentage identity of KW DENV-1 with isolates from different geographic regions.

Strains	GenBank accession number	Year isolated	Identity with KW (%) Nucleotide	Identity with KW (%) Amino acid	
Nicaragua (NI)	FJ562104	2006	99.4		99.7
Mexico (MX)	GU131960	2009	99.0		99.3
Puerto Rico (PR)	EU482591	2006	97.6		99.2
Brazil (BR)	AF311956	1997	97.7		98.9
Argentina (AG)	AY277665	2003	97.0		97.0
China (CN)	JQ048541	2005	91.4		96.8
Western Pacific (WP)	AY145123	2002	92.3		96.9

A previous study reported that NI strains are in the Central American clade, which is a distinct clade from others such as isolates from South America, Caribbean, and Asian Pacific based on phylogenetic analysis with the sequence from the envelope regions [37]. Because many phylogenetic studies showed a consistent clade across continents, one DENV-1 strain from each clade was chosen based on similarity to the KW strain (Table 1) and used in further genomic sequence analyses. When comparing the KW sequence with highly similar sequences, including the NI2006 DENV-1 isolate and other strains representing their clades, eight amino acid substitutions appeared in the envelope and nonstructural protein regions (Table 2). Pairwise distances between the KW DENV-1 strain and other DENV-1 strains were calculated at the nucleotide and amino acid levels (Table 3). Results from this analysis confirmed that the KW strain is less genetically diverse from the NI strain than from the other strains at both the nucleotide and amino acid level, with amino acid sequences from Chinese (CN) and Western Pacific (WP) DENV-1 strains being the most diverse.

**Table 2:** Non-synonymous amino acid mutations that distinguish the KW DENV-1 strain.

Gene	Position	Key West	Nicaragua	Puerto Rico	Brazil	China	Western Pacific
Envelope	484	R (AGA)	K (AAA)	K	K	K	K
NS1	1021	T (ACA)	I (ATA)	I	M (ATG)	I	I
NS2B	1361	L (CTT)	I (ATT)	I	I	I	I
NS2B	1444	V (GTT)	L (CTT)	L	L	L	L
NS3	1578	R (AGA)	G (GAA)	G	G	G	G
NS4B	2488	V (GTA)	L (TTA)	L	L	L	L
NS5	2495	M (ATG)	T (ACG)	T	T	T	T
NS5	3102	H (CAT)	N (AAT)	N	N	N	N

**Table 3:** Pairwise genetic distance of nucleotide and amino acid sequences in DENV-1.

	DENV-1 strains					
Strain	Key West	Nicaragua	Puerto Rico	Brazil	China	Western Pacific
Key West	***	0.009	0.023	0.023	0.094	0.082
Nicaragua	0.005	***	0.020	0.020	0.092	0.081
Puerto Rico	0.011	0.009	***	0.023	0.093	0.083
Brazil	0.012	0.010	0.013	***	0.089	0.078
China	0.041	0.039	0.040	0.040	***	0.076
Western Pacific	0.037	0.035	0.036	0.035	0.030	***

The upper part of diagonal shows nucleotide pairwise genetic distance and the bottom part shows amino acid pairwise genetic distance.

### Genetic Analysis

**Figure 2:**
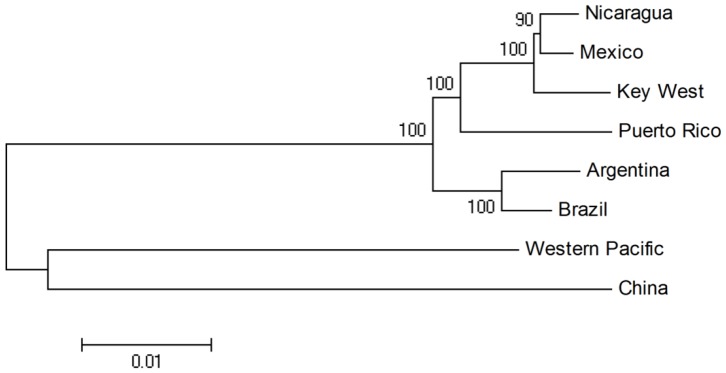
Phylogenetic tree of DENV-1 genome sequences from different geographic regions. This indicates Key West and Nicaragua as offspring strains of Puerto Rico.

**Figure 3:**
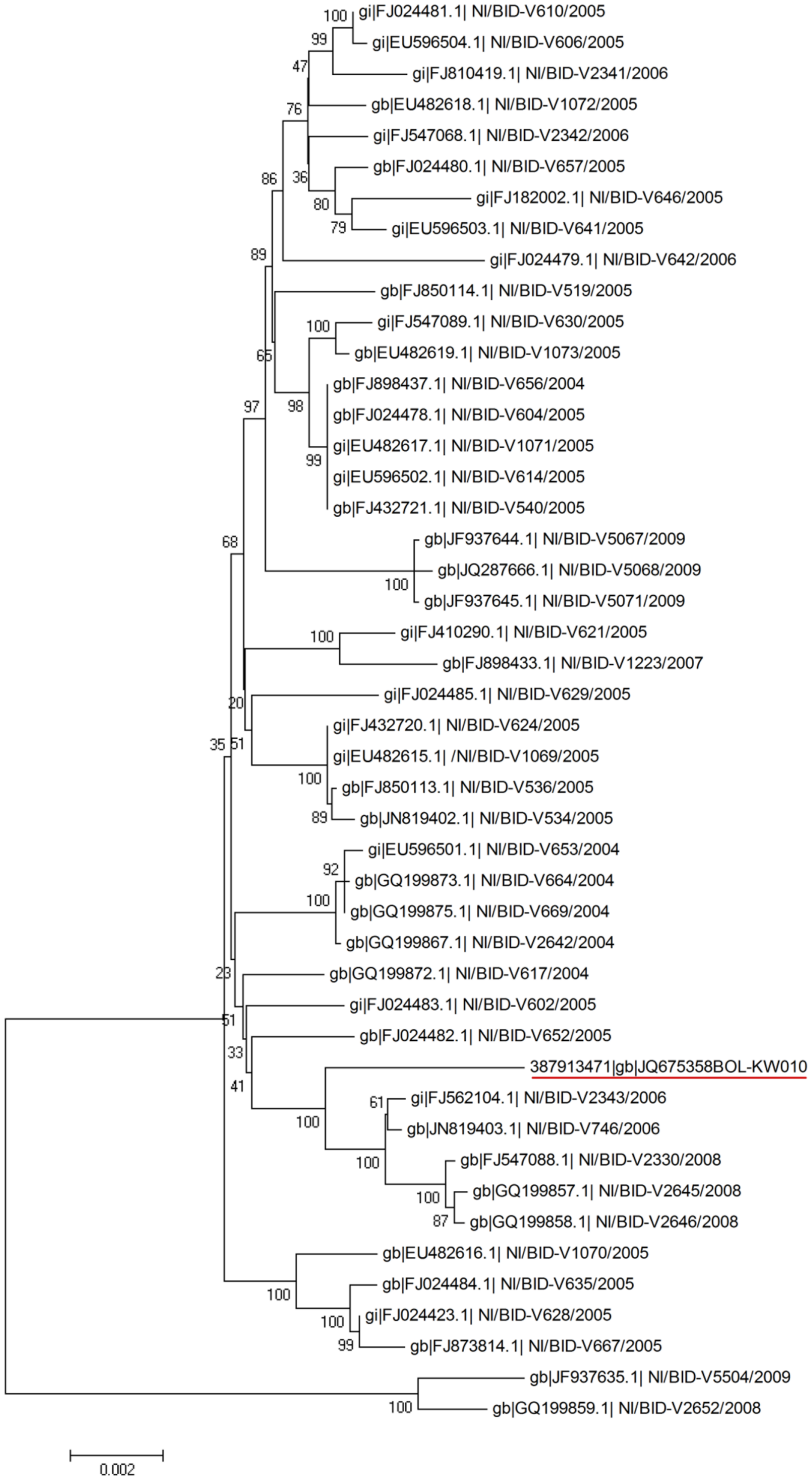
Phylogenetic tree of DENV-1 genome sequences from Key West and 45 different isolates from Nicaragua. Sequences are labeled with GenBank accession numbers|site|isolate name|year and Key West is underlined. KW = Key West; NI = Nicaragua.

The PR strain is the apparent parental strain of NI and KW according to the phylogenetic analysis of complete DENV-1 genomes (Figures 2 and 3). The phylogenetic tree showed results consistent with those from other studies with DENV-1 [5] [37], where DENV-1 strains from Brazil (BR) and Argentina (AG) grouped in South America region and, PR in Caribbean, NI and KW segregated together as members of the Mexico sub region [37]. We selected WP and CN strains as reference sequences to show the relationship between genetic distance and geographic distance.

**Figure 4:**
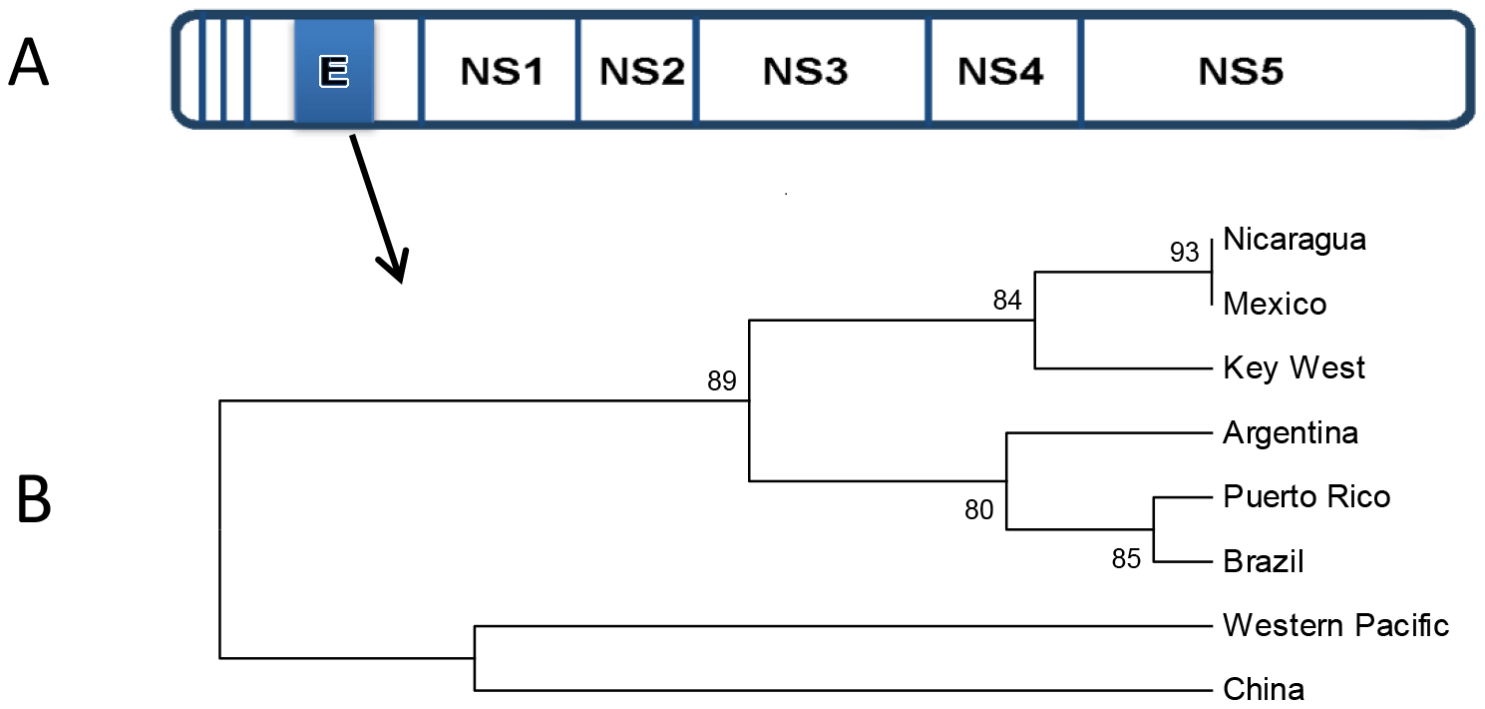
Analysis of recombination events occurring in geographic strains of the DENV-1 viral genome. A . The schematic shows a representation of the genome for the Key West DENV-1 isolate (E = envelope; NS1-5 =  nonstructural regions 1–5). The shaded areas represent the regions where recombination events were identified. **B**. Phylogenetic tree of DENV-1 sequences in the recombination region (E) from geographic strains of DENV-1 clustered PR with BR and AG.

**Figure 5:**
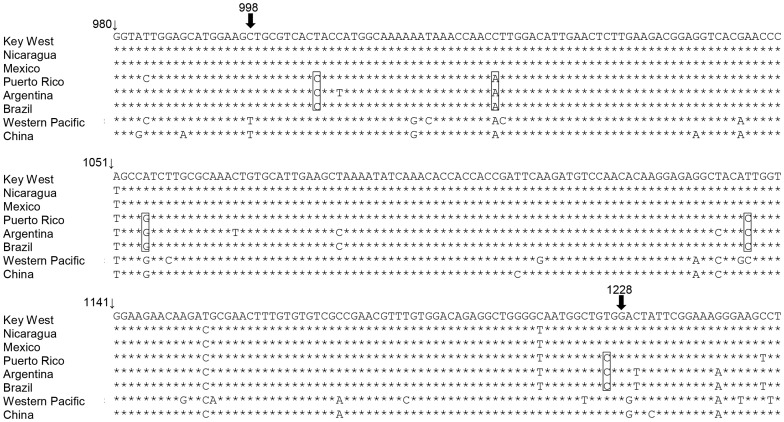
Nucleotide alignment of the recombination region. The breakpoints in the recombination region between KW and PR are indicated with black arrows. The stars represent identical nucleotides between sequences, nucleotides of PR conserved with BR and AG are boxed.

We used the Recombination Detection Method (RDP4) software, which is designed to explore recombination based on statistical analysis between sequences to screen for putative recombination events [38]. The data from RDP analyses suggested that there were recombination events at the same position (nucleotides 997 and 1229 bp) on the KW genome sequence and the location of the event was verified with five different methods. The results from recombination detection analysis are shown in Table 4. The position of recombination is between nucleotides 997 and 1229 of the KW sequence, which is in the envelope region (907∼3453 bp), with *p-*values less than 0.05 in four different analyses and two other analysis detected the same region (nucleotides 997 and 1229 bp) as a putative recombination region *p-*values less than 0.1 (Table 5). The recombination is found between PR and KW among the strains that we analyzed. Comparing two phylogenetic trees of genome and recombination region by recombination analysis showed PR and BR as major and minor parents of KW and NI DENV-1 respectively. Unlike the phylogenetic tree of the genome sequences (Figure 2), PR DENV-1 is grouped with BR (Figure 4) in the phylogenetic tree of the recombination region. The putative recombination region between KW and PR is aligned with other strains of DENV-1 sequences (Figure 5). The boxes around nucleotides represent conserved nucleotides in the putative recombination region but not conserved genome wide (Figure 5).

**Table 4:** GenBank reference sequence information for dengue virus isolates.

Code	Year of isolation	Location	Source	GenBank accession no.
DENV-1 KW	2010	Key West, FL	Human	JQ675358
DENV-1 PR‡	2006	Puerto Rico	Human	EU482591
DENV-2 PR†	2007	Puerto Rico	Human	EU482553
DENV-3 PR†	2004	Puerto Rico	Human	FJ182004
DENV-4 PR‡	1994	Puerto Rico	Human	FJ226067

† Passaged once in C6/36 cells.

‡ Passaged twice in C6/36 cells.

**Table 5:** Recombination Statistics.

Algorithm	Recombination P-value	NT Position
RDP	0.09639	997–1229
GENECOV	-	-
Bootscan	0.09305	997–1229
MaxChi	0.01709	997–1229
Chimaera	0.01152	997–1229
SiScan	0.04050	997–1229
PhylPro	-	-
Lard	-	-

The KW strain has relatively different sequences in the 3′ un-translated region (UTR) of the genome, 10510 bp - 10605 bp (93%), from the NI strains showing the best match at the genome level, although there is no significant difference by recombination analysis. The KW strain retains 99% nucleotide identity with NI2006 strain in the region.

## Discussion

Dengue virus is endemic in PR and there is potential for importation of human cases into FL. Our analyses on virus growth for DENV isolates from PR and FL showed differences in titers between isolates at different dpi for both initial and later growth phases. The KW DENV-1 strain tended to replicate at a higher rate than the PR DENV-1 strain, suggesting a replicative advantage as observed for other dengue viruses [34] [35]. The interaction we observed between isolate and dpi for both the initial and later growth phases indicates that titer differences between isolates changed with dpi. Although definitive evidence is lacking, we have shown that in most instances the KW DENV-1 strain had similar or greater growth than other dengue viruses (serotypes 1–4) from PR, which may, in part, contribute to endemic transmission in Florida. Although DENV growth *in vitro* is unlikely to be quantitatively accurate predictor of growth in primate hosts and mosquito vectors, it serves as a useful indicator of trends in growth in nature. Repeated passages can allow adaptation to growth in cell culture. However, this seems unlikely to explain the observed results given the limited number of cell passages by these dengue viruses.

The full-length genome sequence showed that KW DENV-1 likely originated from NI. The current study will help identify and perhaps mitigate accidental importations of DENV from other dengue endemic and epidemic areas by providing preliminary information about possible import routes. The KW DENV-1 was isolated from a human in October 2010 during rainy and hurricane seasons. Because PR suffered one of its largest dengue outbreaks in 2007, with all four serotypes (DENV-1, 2, 3, and 4) circulating [10] [11], we hypothesized that the PR strain carried by tourists was a candidate progenitor for the KW DENV-1. However, our findings indicate that the KW strain is more closely related to the NI strain than the PR strain based on the sequence similarity and genetic distance (Figure 2), suggesting that the KW strain originated from NI as a member of the Central America Clade. The PR strains belong to the Caribbean Clade and the Central America, South America and Caribbean clade share the same ancestor from the Asian Pacific group [37]. Our findings indicate the KW and NI strains are offspring strains from PR.

The envelop region of PR DENV-1 strain used in this study is 100% identical with DENV isolated from Venezuela, collected from 2005 through 2008 based on NCBI Blast analysis, showing that the sequences of the envelop region are maintained through generations. Also, KW DENV-1 showed 100% identity to the DENV-1 envelope sequences published in Graham et al. [37] and this sequence is derived from DENV-1 isolated from mosquito samples collected in KW. According to Weaver and Vasilakis [39], recombination in virus strains can be confirmed if the genomic sequences used in the analysis fulfill a few conditions. Those conditions are that the sequence of the identified recombinant must be from a single PCR amplicon, the recombinant must be reproducible in clonal populations, and the recombination event must be maintained through future generations. Because the PR strain sequences used for the study were downloaded from GenBank, information about how they were sequenced is not known; however, the recombination region identified in the KW and PR strains seems to be conserved and has been maintained in these populations of DENV-1 for years. The recombination region would not have been maintained without reproducing the region in the clonal populations and single PCR amplicon sequencing is required to avoid patch walking from sequences of different viral isolates. In fact, the use of the third criterion to confirm recombination covers the other two requirements. The sequence of the KW DENV-1 strain was also generated from a single PCR product of the region (primer set: forward CCGAAACATGGATGTCCTCT reverse TCTCCCATTCTGGGTCACTC) used in the analysis and its comparison to sequences of this region from other isolates showed a high degree of similarity. Thus, the recombination in PR and KW strains appear to meet the requirements for being a recombination event.

Nicaragua experienced a DENV-1 outbreak from 2002–2005 and 45 variant genome sequences are available in GenBank (2004–2009). The phylogenetic analysis of the envelope region by Graham et al. [37] showed some NI strains are closely related to the KW strain, which is the same result found in this study (Figures 2 and 3). Because the KW strain is closely related to the Central American clade, based on genetic distance, and NI and MX are geographically close, it is possible that the NI and MX strains circulated together or that one strain was transported to the other region via infected mosquitoes or tourists and subsequently introduced into FL.

The KW DENV-1 outbreaks occurred from 2009–2010 and there were several sporadic dengue cases in the Florida peninsular region between 2010–2012, although there is no genome sequence data currently available from the 2009 and 2011 DENV-1 isolates. There was a dengue outbreak in Texas in 2006, speculated to have come from neighboring MX [16]. The DENV isolate from the Texas outbreak also has not been propagated as a stock and no sequence data is available to date. Regardless of whether the KW strain came through MX, specifically from the Texas outbreak, or directly from NI, the KW strain has evolutionary differences from the sequences showing the highest similarity and this might suggest that KW has its own circulating DENV-1 strain that originated from NI. Additional studies are needed to assess whether genetic differences between the NI and KW strains translate to differences in interactions between vectors (e.g., vector competence for DENV), hosts, environmental and/or other factors. The NI strains from 2004 to 2008 were the best matched sequences to the KW DENV-1 strain, followed by the MX strains. In addition, phylogenetic analysis with 45 of the NI strains (2004–2009) and the KW strain genome sequence (Figure 3) clustered the NI strains by years and clustered the KW strain in a different subgroup from the NI 2009 strains, suggesting that the KW strain was introduced before 2009. Interestingly, the branch of the KW DENV-1 strain in the phylogenetic tree is not joined with any other strains and the 3′ UTR region was different from the NI strains. Thus, the KW DENV-1 strain seems to have originated from one of the NI strains but has, during a short period of time, become genetically distinct, perhaps owing to different selective pressures associated with translocation to a new geographic region [40]. Our study suggests that the KW strain could be more isolated from the MX and NI strain geographically, since there is no recombination event between the NI and MX DENV-1 strains and their genetic distance is closer than to KW (0.004: 0.009) at both the nucleotide level and amino acid level (Table 3). Also, with the same reason that NI and MX are almost identical, the MX is not included in Table 4. The relatively different sequences in the UTR between KW and NI does not confirm the recombination of KW and NI. We speculate that the lack of evidence for recombination in the UTR between KW and NI may be, in part, attributed to the short time of their translocation, and so may obscure the detection of recombination events.

Hence, it is plausible that the KW strain was introduced into KW before 2009 and circulated in that region between 2009 and 2010 [17] [18], and this is evidence that the KW strain is different than the NI strain. The difference may not be due to evolutionary recombination but could be the result of adapting to the local environment in the United States. Since no effective drug or vaccine is available for dengue and international tourism can spread the disease agent, the possibility of local DENV circulation could become a more widespread threat to public health in the United States. Our results suggest that the possibility of the presence of a distinct DENV strain in KW merits careful consideration.

## Materials and Methods

### Viruses and Culture Conditions

Dengue virus (DENV) isolates were obtained from the Centers for Disease Control and Prevention (DENV-1-4 from PR) and Florida Department of Health, Bureau of Laboratories (DENV-1 from KW, Florida) (Table 4). Dengue viruses from PR underwent 1–2 passages on mosquito *Aedes albopictus* C6/36 cells followed by a single passage on African green monkey kidney (Vero) cells (American Tissue Culture Collection, Manassas, VA) prior to use in this experiment (Table 4). Dengue-1 virus from KW, Florida was isolated from a human in 2010 and passaged twice in Vero cells prior to use in the growth experiment. Viruses and host cells were cultured in media containing medium 199 with Earle's salts, 10% fetal bovine serum, 0.2% amphotericin B and 2% penicillin/streptomycin. Cells used for infection were Vero cells. Cells were grown in 75 cm^2^ tissue-culture flasks at 35°C and 5% CO_2_ atmosphere to achieve confluent monolayers consisting of ∼7.4×10^6^ cells. For each isolate, a cell monolayer was infected with 0.4 mL of media containing DENV at a multiplicity of infection of 0.0001 virions per cell. Four replicate flasks containing monolayers were used to amplify each virus population. After 1 hour incubation at 35°C and 5% CO_2_, 12 mL media was added to each monolayer of cells. The infected monolayers were then returned to an incubator set at 35°C and 5% CO_2_. The next day, 1 mL of supernatant was sampled from each virus population and replaced with 1 mL media. This process was repeated daily for 12 days between 1000 and 1300 hours. At each sample period, aliquots of supernatant containing the virus progeny drawn from each virus population were frozen at −80°C for future analysis.

### Determining Growth rate of DENV

Titrations of DENV were performed by plaque assays in 6-well plates containing monolayers of Vero cells as described elsewhere [41]. Briefly, serially diluted samples were plated in single wells using 0.25 mL inoculums on Vero cells under 2X Eagle's Minimum Essential Medium containing 9% FBS, 1% L-glutamine, 2% non-essential amino acid solution, 2% Amphotericin B, 0.2% penicillin/streptomycin and solidified with 1.6% Seaplaque® low melting agarose. Plaque assays were incubated for six days at 35°C and 5% CO_2_ atmosphere followed by a second overlay consisting of distilled water, 1% sodium chloride and 4% neutral red solution solidified with agarose. The infected monolayers were incubated at 35°C and 5% CO_2_ for another 24 hours after which plaques were enumerated and expressed in plaque forming units/mL (pfu/mL). Each plaque was assumed to have originated from a single infecting virion.

We conducted two separate statistical analyses for virus titers (i.e. 1–5 days post-inoculation (dpi) and >5 dpi) based on visual inspection (Figure 1). The growth rate increased between 1–5 dpi and generally plateaued >5 dpi. No data were collected for the DENV-1 (KW) for 9 dpi. Analysis of variance (PROC GLM, SAS 9.22) was used to evaluate differences in virus growth kinetics for the DENV isolates, dpi, and isolate x dpi. If significant differences were observed, then a Duncan test was used to determine differences in the means (Figure 1).

### RNA extraction and cDNA synthesis for amplification and sequencing

Viral RNA was extracted from the supernatant of infected cells, previously infected with one isolated KW DENV-1 plaque, using Trizol LS Reagent according to the manufacturer's instructions. cDNA synthesis and amplicons of the target sequences were carried out using the Enhanced Avian HS RT-PCR kit (Sigma, Saint Louis, MO) with reverse transcription completed using random nonamer, generating cDNAs of various sizes. Gel electrophoresis was used to confirm the size of the target PCR product. The PCR product was purified and sequenced using the Sanger dideoxy sequencing method with Genomelab DTCs-quick start kit from Beckman Coulter [42].

### Primer design

A total of 38 synthetic oligonucleotide primer pairs were designed to amplify overlapping PCR fragments of size ranges between 500–600 bp spanning the whole genome of DENV-1 viral RNA. All primer sets were designed and ordered through Integrated DNA Technologies, Inc (Coralville, IA). The first 8 sets of primer sequences were designed based on the full-length sequence of the PR DENV-1 strain. Other primer sets were designed based on sequencing results of the KW DENV-1 strain and all the primers were located in the middle of the sequence of the previous PCR product to generate overlapping sequences. The sequencing was conducted at least 4 times per primer and in both directions.

### Genome sequence assembly and genetic analysis

The complete genome sequence of the KW DENV- 1 isolate was sequenced, assembled with DNASTAR Version 10.0.0, and deposited in the GenBank database under accession number JQ675358. The KW sequence was compared with other DENV-1 strains, from Nicaragua (NI), Puerto Rico (PR), Brazil (BR), China (CN), and Western Pacific (WP), in aspects of genetic distance determination and neighbor-joining phylogenetic tree building, using DNASTAR version 10.0.0 (Figure 2). We added sequences from Mexico (MX) and Argentina (AG) in the recombination analysis and phylogenetic analysis to enhance the illustration of their evolutionary relationship.

Systemic recombination analysis was carried out with Recombination Detection Program (RDP4 beta version 4.16) software [43] for statistical identification and characterization of historical recombination events with a set of aligned nucleotide sequences [38]. The software includes RDP, GENECOV, BootScan. MaxChi, Chimaera, Siscan, PhylPro, LARD, and 3Seq methods and has previously been used in analyzing recombination among various virus species including West Nile virus [44]. There was one recombination event detected by RDP and BootScan with *p*-value <0.01 and the same recombination region was detected by three other methods with *p*-value <0.05. Their recombination *p*-value (*p*-value <0.1.) by RDP and BootScan might be considered relatively low. However, the sequences used in this analysis are highly similar and genetically closely related thus, we accepted their *p*-value. Parameters were applied in 50 bp window size of scanning consensus sequences and recombination rate was identified between sequences sharing 82% to 92% of similarity (Table 4). The detected recombination region in the KW DENV-1 in this study is shown in Figure 5, aligned with 7 other different DENV isolates.
